# Leadership as a determinant of need fulfillment: implications for meta-theory, methods, and practice

**DOI:** 10.3389/fpsyg.2024.1427072

**Published:** 2024-06-25

**Authors:** J. David Pincus

**Affiliations:** Leading Indicator Systems, Boston, MA, United States

**Keywords:** leadership, transformational leadership, servant leadership, sustainable leadership, toxic leadership, organizational culture, employee engagement, employee well-being

## Abstract

Of all the most prominent business concepts (e.g., DE&I, employee well-being, employee engagement, organizational culture, etc.) none rivals *leadership* in terms of public interest and annual monetary investment. Despite the obvious importance of leadership as a determinant of many important outcomes, the concept of leadership has been surprisingly hard to pin down, lacking consensus as to its precise meaning. As numerous authors introduce ever more constructs (e.g., servant leadership, toxic leadership, sustainable leadership, transformational leadership, etc.), the leadership concept has become emblematic of the problem of construct proliferation. Like the related fields of employee engagement, subjective well-being, and organizational culture, the leadership field is in desperate need of a clearly articulated meta-theory to house its many constructs, allowing theory and measurement to *build* up instead of continuing to *pile* up. This paper argues for grounding the concept of leadership within the psychological literature on human needs. In reviewing the leading definitions of leadership in the literature we find that they are reducible to a core set of follower needs that can be facilitated or inhibited by leaders. We propose that there is substantial value in adopting a comprehensive needs-based taxonomy over current approaches. We consider the impact of setting the concepts of leadership within existing need constructs for each of the following: (a) theory, especially the development of leadership frameworks and particularly how the concept of leadership relates to the concepts of organizational culture, employee well-being, and employee engagement; (b) methods, including the value of applying a comprehensive, structured model; and (c) practice, where we emphasize the practical advantages of clear operational definitions.

## Introduction

The leadership concept has remained a primary focus in both the private and public sectors, representing the single largest human resources expenditure ($356 billion in 2015; Beer et al., 2016) outside of salary and benefits. Year after year, the most popular category of business books is leadership skills and effective management. Strong and growing recent interest in this concept is confirmed by Google Trends (Accessed May 22, 2023), which shows an upward trend in Google searches of “leadership” from an index low of 62 in April 2018, increasing to an index of 100 by April 2023, indicating the strongest search volume to date. This astounding level of interest persists despite serious questions about the return on investment associated with leadership skills training as evidenced by shockingly weak global levels of employee engagement,^1^ and indeed, questions about the very definition of the concept itself. A recent Segal (2021) article reported that only 11 percent of companies rate themselves as having a strong leadership team, the lowest rating in a decade. Clearly, something is wrong with leadership, yet without a clear notion of what leadership is, its key dimensions and elements, it becomes extremely difficult, if not impossible, to adequately teach the necessary skills. This paper argues for a more grounded approach to the concept of leadership, setting it in the broad psychological literature on human needs.

### The current state of theory

Recent literature reviews of leadership theory and measures have concluded that as a consequence of the growing popularity of leadership studies in both academia and applied settings, concepts have wildly proliferated in violation of Occam’s razor (Banks et al., 2018). Banks et al. (2018) conducted a sweeping audit of 57 meta-analyses, representing several decades of leadership theory, to find “alarmingly high” intercorrelations among constructs, suggesting that the field suffers from a complete lack of focus on parsimony as a goal. As suggested by several academic observers of this field, unchecked concept proliferation endangers the entire body of leadership theory and practices derived from it (Le et al., 2010; Shaffer et al., 2016; Antonakis, 2017). “The fragmentation of research in different, largely non-communicating parts of the literature… prevents studying leadership behavior in a manner that covers the comprehensiveness of leadership” (van der Hoek et al., 2021, p. 375).

“Extensive research on leadership has given rise to many leadership theories and models… yet no universal definition of leadership is agreed upon.” (Asrar-ul-Haq and Anwar, 2018, p. 179).

“There has been a bewildering proliferation of taxonomies on leadership behavior… different terms have been used to refer to the same type of behavior… the same term has been defined differently by various theorists… it is difficult to translate from one set of concepts to another.” (Yukl et al., 2002, p. 15).

“New leader behavior theories continue to be conceived without explicit comparison to, or falsification of, existing leader behavior theories.” (DeRue et al., 2011, p. 15).

We will now briefly summarize some of the major theoretical approaches to give the reader a sense for the diverse and overlapping distinctions currently in use.

#### Transactional vs. transformational

The Transactional-Transformational leadership distinction continues to be influential partly due to the popularity of the Multifactor Leadership Questionnaire, which was designed to measure these constructs (Batista-Foguet et al., 2021; Bajcar and Babiak, 2022). Originally introduced by Bass and Avolio (1993), it differentiates between “economic” relationships based on leader-follow exchange dubbed Transactional Leadership and a style of leadership that involves inspiring and motivating followers by setting a clear vision, fostering creativity and innovation, and encouraging personal growth and development called Transformational (or Visionary) Leadership.

Transactional Leadership is equated with the principle of Contingent Reward, wherein leaders establish performance goals, define incentives that will be provided in exchange for meeting them, and determine if, and how much, incentive will be paid. Two sub-types were offered by Bass and Avolio: Management by Exception – Active: Contingent Reward with active monitoring of performance and feedback to ensure that employees remain on track; and Management by Exception – Passive: reactive Contingent Reward, i.e., without active monitoring or feedback unless problems reached a critical threshold. As an indicator of the conceptual slipperiness abounding in this field, the leadership literature typically refers to these as *three* distinct types (or, worse, *dimensions*, or worse still, *paradigms*) of Transactional Leadership, when it is apparent that we are talking about a single scheme (economic exchange) accompanied either by fast (active) or slow (passive) performance feedback.^2^

Transformational Leadership was described by Avolio et al. (1991) as coming in four distinct types: Idealized Influence, leadership that gains the trust and admiration of followers; Inspirational Motivation, leadership based on inspiring followers through provision of a compelling vision and sense of purpose; Intellectual Stimulation, leadership based on creativity, innovation, and critical thinking among followers; and Individualized Consideration, leadership based on genuine concern for the needs, interests, and development of individual followers. Here, even within a single theory, we begin to see conceptual difficulties emerging. Beyond active vs. passive transactional leadership, none of these concepts is mutually exclusive, or represent orthogonal dimensions. A leader who values critical thinking and innovation will typically gain trust and admiration, just as a leader who provides a sense of mission will usually show concern for the needs of individual employees. In fact, we would fully expect leaders who provide any of the four transformational styles to also provide the others to some degree because they are not independent.

#### Further conceptual elaborations

Emblematic of theoretical development in the leadership field, the Bass and Avolio model was expanded upon by Avery (2004) who kept the Transactional-Transformational distinction but added Classical (i.e., autocratic) and Organic (i.e., democratic).

A conceptually similar set of distinctions have been proposed by Goleman (1995) positing six types of leadership defined by differences in emotional intelligence: Coercive style tends to be autocratic and commanding; Authoritative style mobilizes followers on the basis of the leader’s vision akin to the Avolio et al. (1991) Inspirational Motivation concept; Affiliative style brings followers together with a sense of belonging; Democratic style relies on follower input to set direction; Pacesetting style attempts to motivate followers by setting the example of the leader’s own high standards for themselves; Coaching style focuses on the development of followers in building their strengths and therefore takes a longer-term perspective, and bears a resemblance to Avolio et al. (1991) Individualized Consideration concept.

This approach of adding conceptually overlapping leadership styles over time is emblematic of the field’s struggles with concept proliferation.

#### Unit of analysis: leaders or leadership

A major debate has focused on the relative merits of the traditional study of leaders vs. the dynamic study of leadership. The leader school focuses on the individual traits and behaviors of leaders themselves, whereas the latter addresses broader processes and interactions that define leadership as a dynamic phenomenon. The key difference lies in the individualistic versus systemic perspectives: the former views leadership as a quality or capability of specific individuals, while the latter sees it as a collective process that is distributed, emergent, and context-dependent (Higgs, 2022; see Discussion/Limitations below).

#### From inspiration to ethics

In the wake of a series of crises of leadership, including the accounting and credit ratings scandals of the early 2000s, along with a resurgence of nationalism and extremism, the way leadership is conceptualized has broadened substantially from traits and behaviors that inspire worker productivity to a more global, Aristotelian concept of moral virtues (e.g., honesty, integrity, and ethical conduct). There is no shortage of such new definitions of leadership, which go by names like servant leadership, ethical leadership, altruistic leadership, authentic leadership, shared leadership, and spiritual leadership (Banks et al., 2018).

#### A call for a pause

Recognizing the current strong degree of concept proliferation, Banks et al. (2018) have called for a moratorium on new leadership concepts “until we are able to cumulatively integrate what we have so far theoretically” (p. 247). These authors state the need directly: the field needs a “grand unified theory of leadership” (p. 246) that can explain outcomes like job performance and organizational citizenship behaviors while integrating concepts as diverse as trust, purpose, empowerment, relational quality, authenticity, ethics, and fairness. It is this call that we hope to answer with this paper by attempting to organize the myriad leadership factors within a comprehensive framework of universal human emotional needs or motivations.

#### Why motivation?

We will argue that leadership is fundamentally a relational, social phenomenon that dynamically interacts with individual humans and teams of humans for the purpose of meeting specific human requirements. This assumption is explicit in the literature on servant leadership, which emphasizes that a leader’s role should be first and foremost in the service of meeting follower emotional needs for things like well-being, growth, ethics, community, and higher purpose. Across every domain of human endeavor, effective leadership aims to fulfill the needs of followers directly or indirectly, from the most basic needs for physical and psychological safety, to facilitating personal growth and material wealth, to providing inspiration and a higher purpose. This is as true of the team leader of a tech product development team as the leader of a nation. In this light, we can see that leadership concepts emerge to characterize specific situations defined by specific sets of follower needs, which continue to evolve as the leader-follower contract becomes ever broadened and more holistic.

#### Foundational vs. aspirational needs

The popular distinction between transactional and transformational leadership styles can be seen as an attempt to distinguish between a focus on lower, more basic needs by establishing clear rules governing safety, degree of autonomy, and fair distribution of rewards (i.e., transactional) vs. a focus on evolving mechanisms to meet higher, aspirational needs for self-actualization, esteem, and transcendent, ethical purpose (i.e., transformational).

#### Need categories

Diverse leadership concepts can similarly be viewed as addressing different categories of human needs. Servant leadership, for example, tends to concentrate on needs of the self for psychological safety, authenticity, and personal growth, as well as social needs for belonging, empathy, and esteem. Ethical leadership, on the other hand, is squarely focused on spiritual needs for fairness, justice, ethics, and higher purpose. This same logic will be applied to a wide variety of leadership theories in this paper.

#### Benefits of anchoring leadership concepts in human needs

By anchoring the diverse theories of leadership to their underpinnings in specific human needs, it becomes possible to vastly simplify and organize leadership concepts according to the needs they fulfill. This elemental approach not only makes the varied leadership literature easier to understand and teach, but also provides a practical framework for leaders to choose an appropriate style based on the needs of their followers. By clearly connecting the disparate surface features of leadership concepts to their purpose and function in meeting specific follower needs, we hope to break the current conceptual “log jam” and allow a more streamlined understanding of leadership theory and practice.

## Method

### Literature review

In accordance with the six-step procedure offered by Templier and Paré (2018), a literature review of leadership theory was conducted consistent with the six-step process outlined by these authors: (1) problem formulation, (2) literature search, (3) screening for inclusion, (4) quality assessment, (5) data extraction, and (6) data analysis and interpretation, as follows:

(1)  The primary goal of this review is to identify theoretical systems that purport to define the components of leadership.
(2)  The literature search was performed using multiple, iterative search strategies beginning with consultation of the Web of Science and Google Scholar search engines, using combinations of keywords drawn from definitions of leadership taking the form of “leadership” modified by the following kinds of terms: “effective,” “charismatic,” “*laissez-faire*,” “abusive,” “team,” “servant,” “relational,” “trait,” “transactional,” “transformational,” “altruistic,” “ethical,” “authentic,,” “shared,” “spiritual,” “classical,” “organic,” “inspirational,” and “visionary.” As relevant papers were identified, the list of search terms was updated to include additional terms. The following terms were added during this process: “participative,” “strategic,” “democratic,” “cross-cultural,” “situational,” “coaching,” “narcissistic,” “inclusive,” “toxic,” “resilient,” “innovative,” and “values-based.”

Further backward and forward searches on relevant papers permitted the discovery of additional materials.

(3)  The searches described above resulted in a vast number^3^ of publications of multiple types, which were further screened for inclusion. Screening criteria focused on the presence of a comprehensive model of leadership, whether viewed through the lens of psychology, sociology, management, or assessment. Additionally, results were screened for the availability of a complete set of assessment items that corresponded to each comprehensive model. Screening the results for the presence of terms “theory, “model,” “factors,” “dimensions,” “assessment,” and “measures” reduced the set further.
(4)  At this point, the full set of publications were reviewed for quality and relevance, resulting in additional forward and backward searching, which revealed a final set of conceptual models that conformed to the above requirements.
(5)  The specific elements of each model were extracted into a table for direct comparison (Supplementary Table S1).
(6)  The analysis and implications are presented below.

The literature review revealed the existence of several previous literature reviews that have partially cataloged theory-driven leadership concepts (Shaffer et al., 2016; Banks et al., 2018; Lemoine et al., 2019; Rudolph et al., 2020). We conducted a more exhaustive literature review to identify a fuller set of concepts and items. We examined concepts, whether described as factors, themes, dimensions, etc., as evidence of the primary meaning intended. We also examined specific measurement items designed to represent each concept as a further attempt to reveal the intended meanings of concepts.

The analysis resulted in the identification of 50 unique non-trait concepts and 267 individual assessment items discovered in the literature review (Supplementary Table S1). With the search completed, we employed the five-step procedure suggested by Dwertmann and van Knippenberg (2021) for conducting an integrative review: (1) define the review topic and search strategy [completed in accordance with Templier and Paré (2018), as described]; (2) code studies in terms of an initial theory-based set of attributes and determine how well this captures similarities and differences in findings; (3) code studies based on attributes drawn from theory outside the review area or derived inductively to capture similarities and differences not predicted by theory in the area of review; (4) propose new theory integrating theory-inconsistent findings anchored on the step 3 categorizations; and (5) determine an agenda for future research anchored in the theoretical integration.

### Coding components and items according to initial theory

Following Dwertmann and van Knippenberg (2021), we coded components and items in terms of their original theoretical categories and determined how well these categories made sense of the assembled components and items. Supplementary Table S1 contains all elements drawn from the literature review, both individual assessment items and the components or factors they are intended to represent based on the initial leadership theories from which they are derived. To answer the question of how well these theories capture similarities or differences of core concepts, we quote two recent conclusions on the state of this literature:

“Our review of the leadership literature suggests that numerous scholars have voiced concerns over the extent to which construct proliferation has crept into this area of study.” (Shaffer et al., 2016, p. 93).

“In sum, the leadership literature appears to be both in need of and ready for parsimony.” (Banks et al., 2018, p. 237).

“Insomnia? Try counting leadership theories” (Higgs, 2022, p. 355).

Our analysis lends additional support for the conclusion that leadership theory is multidimensional and complex but not well organized. Leadership constructs range broadly across conceptual categories from global evaluations of leadership outcomes (e.g., *Overall, to what extent is the supervisor performing his/her job the way you would like it to be performed?*) to the structural processes that deliver these outcomes (e.g., work schedules and assignments) to personality traits (e.g., rigidity, extraversion, social influence), cognitive states (e.g., planning orientation, attentiveness to what’s going on), emotional states (e.g., helps me heal emotionally, expresses concern for others’ feelings), and ethical standards (e.g., altruism for the sake of the team, playing a moral role in society).

At the most abstract level, we encounter what we will call *general evaluations of leaders.* These include components such as *leadership performance against expectations overall, satisfaction with leadership,* and *liking of leaders.* At the next level of specificity, we find a broad array of concepts and assessment items. These range from the personal (e.g., level of anxiety, “blowing up” when overwhelmed, degree of openness to new ideas, striving for personal growth and mastery) to the social (e.g., maintaining a close-knit group, caring for the personal welfare of employees, eliciting feelings of admiration and respect from employees). Concepts similarly span the domain of the tangible and material (e.g., allowing workers freedom to make decisions, pushing for efficiency and productivity, focusing on achievement and accomplishment) to the domain of principles and ideals (e.g., ensuring fair treatment, considering moral dimensions of decisions, emphasizing a higher purpose). With such diverse “raw material” it is little wonder that the field has struggled to define a meta theory to contain and organize these concepts.

### Coding components and items according to theory outside the review area

Following Dwertmann and van Knippenberg (2021), we supplemented the components and items with a new categorization borrowed from a different theoretical domain and determined how well these new categories made sense of the same set of components and items. We argue that beneath these summary level constructs lie the operations of a set of fundamental human needs as described by a recent unified model of human needs (Pincus, 2022a,b, 2023a,b, 2024a,b). This chain of logic proceeds as follows: Leadership concepts tend to reflect the dynamic relations between leaders and followers (Uhl-Bien et al., 2007; DeRue and Ashford, 2010; Higgs, 2022). Relational leadership approaches are fundamentally different than the trait-based school of leadership because relationships are necessarily dynamic across a range of different leader and follower states, whereas the trait perspective holds the leader’s attributes constant regardless of specific followers or situations. It is possible, and we would argue, advantageous, to shift our thinking about leadership from static characteristics of the leader himself or herself (Zaccaro, 2007) toward the dynamic effects produced by the leader in followers (which reciprocally affect the leader).^4^We recently published a review of the concept of employee engagement, which similarly suffers from concept proliferation, and demonstrated that the components of engagement are reducible to a set of core human needs. When these needs are met, employees become engaged; when they are not met, employees become disengaged. We propose that a meta theory of leadership can be constructed to correspond directly to these sets of follower needs; in this approach, leader actions may be seen as either promoting or inhibiting the fulfillment of follower needs. By shifting focus from leader-centric “sender” traits to follower-centric “receiver” states, the wide array of leadership concepts finds homes in a structured model of human needs. For this reason, we have restricted our analysis to non-traits concepts, which include behaviors, emotional states, motivational states, social relations, intentions, expectations, etc.

### Propose new theory to better account for the components and items

Repeated calls have been made for theorists to identify a larger framework for leadership that can integrate the disparate and growing collection of constructs. In keeping with the suggestion of Dwertmann and van Knippenberg (2021), we have applied a structured model of human needs to the set of components and items identified in the literature review and find a strong degree of fit. All of the non-trait concepts identified in our literature review reflect leadership’s degree of support for the fulfillment of discrete human needs, from feeling psychologically safe in the organization to inspiring employees with a higher purpose. These concepts address the domain of the self (e.g., safety, authenticity, potential); the material domain (e.g., autonomy, immersion, success); the social domain (e.g., inclusion, caring, recognition); and the spiritual domain (e.g., justice, ethics, transcendent purpose).

These essential attributes of leadership are strongly aligned with the concept of emotional needs or motivation, defined by Pincus (2004) as *an unobservable state of emotion or desire operating on the will, causing it to act*. The strong alignment between these concepts is rooted in leadership’s need fulfillment function, which interacts with follower motivational-emotional states. The goal of this paper is to suggest that a meta-theory of human needs can accommodate virtually all the wide-ranging components of leadership.

Recently, Forbes (2011) and Pincus (2022a) have introduced a comprehensive taxonomy of human motivations. While numerous “mini theories” of motivation have been proposed in the psychological literature, there has been an enduring absence of a comprehensive system (based on fundamental principles) to categorize motivations such as the needs for achievement, competence, relatedness, immersion, justice, ethics, purpose, or autonomy. Although Maslow’s (1970) need hierarchy has been frequently referenced in the leadership literature (Avolio et al., 1988; O'Sullivan and Adair, 1995; Goleman et al., 2002; Jung et al., 2003), it is incomplete for the present purposes. As a result of Maslow’s focus on atypical, self-actualized individuals, his model inadvertently overlooks a broad range of now-recognized fundamental motives. These include the need for caring identified by Bowlby (1999) and Bowlby and Ainsworth et al., (1965), the needs for material power and achievement proposed by McClelland et al. (1953) and McClelland (1975), the need for experiential immersion (i.e., *flow*) proposed by Csikszentmihályi (1990), the need to form and express one’s unique identity proposed by Erikson (1963), the need for justice described by Bloom (2014) and Lerner (2003), and the need for a moral code described by Kohlberg (1973), Haidt (2008), and Greene (2014).

Our taxonomy is based on first principles of four life domains and three levels of striving. Because motivation always involves a change of state, the taxonomy asks two questions:

1. First, *in what part of your life do you seek change?* The answer to this question is found in one of four life domains: the *self*, the *material*, the *social*, and the *spiritual*. Note that these represent pairs of opposites: self vs. social, and material vs. spiritual. These four domains of human life have been postulated in a variety of fields, including philosophy, psychology, and each of the five major world religions (Pincus, 2022a).
2. The second question is *what level of change do you seek*? To answer this question, we employ Aristotle’s (1933) three states of existence, the foundational level of potential (*being*), an intermediate level of potentiality-as-such (*doing*), and a higher level of actuality (*having*).^5^

By combining the three modes of existence with the four life domains, a comprehensive matrix comprising 12 cells is formed, as there are no additional life domains or modes of existence. In our previous examination of the literature on motivation (Pincus, 2022a), we identified over 100 distinct motivational constructs, all of which were classified into one of the 12 matrix categories of motivation. This observation supports the claim that the matrix is all-encompassing (Table 1). As mentioned, the columns of the matrix represent the four domains of human activity (self, material, social, and spiritual), while the rows represent the desired level of change (foundational, experiential, aspirational).

**Table 1:** A unified pyramid of human motivation (Pincus, 2022a).

Three levels of striving	Four life domains			
	Self	Material	Social	Spiritual
Aspirational	Fulfilling potential and limitation	Success and failure	Recognition and scorn	Higher purpose and materialism
Experiential	Authenticity and conformity	Immersion and stagnation	Caring and uncaring	Ethics and wrongdoing
Foundational	Safety and insecurity	Autonomy and disempowerment	Inclusion and exclusion	Justice and injustice

**Figure 1:**
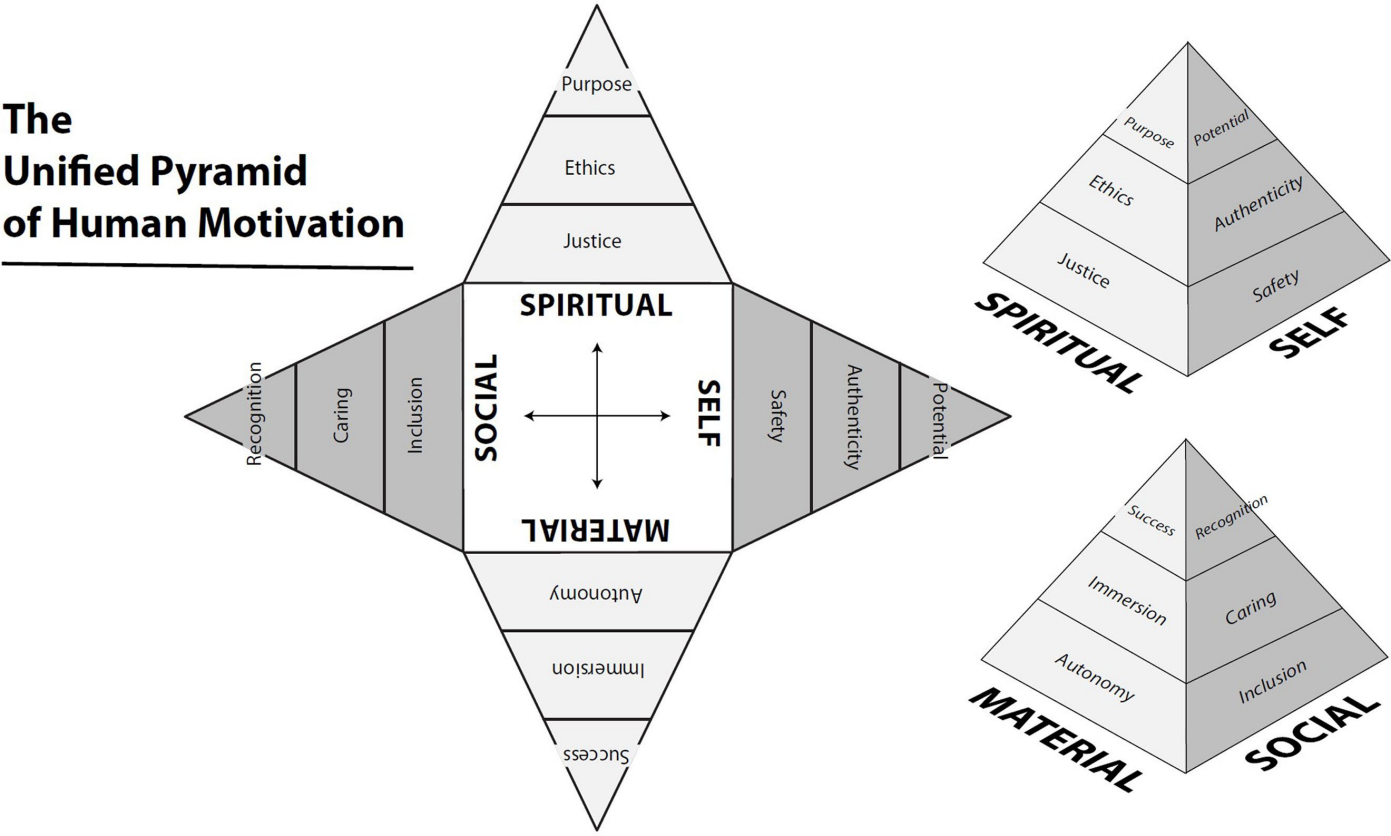
The Unified Pyramid of Human Motivation.

For publication, the matrix is presented as a two-dimensional table (Table 1). However, a more accurate representation would be a three-dimensional pyramid with four sides, as depicted in Figure 1 (Pincus, 2022a). Each face of the pyramid corresponds to one of the life domains. The narrowing from the base to the peak on each side emphasizes the notion that we must start at the foundational level within each domain before progressing toward higher needs. Consistent with Maslow’s (1970) theory, fewer individuals are capable of reaching the higher levels, resulting in their reduced representation toward the apex. The choice of a four-sided pyramid also serves to highlight the opposing nature of the domains, with the self-domain being antipodal to the social domain, and the material domain antipodal to the spiritual domain. This proposition carries implications for generating hypotheses, which we will revisit toward the end of this paper.

The matrix holds two additional features that have implications for leadership. These features pertain to need hierarchies within each life domain and the dynamics of motivational energy, colloquially known as “pull” and “push”:

- Drawing from the principles of Aristotle (1933) and Maslow (1970), our model proposes a hierarchical and temporal sequence. Advancing from foundational to experiential needs or from experiential to aspirational needs necessitates the partial fulfillment of more fundamental needs. Satisfaction of lower-level needs allows higher-level needs to gain salience in driving behavior.
- Each of the 12 needs can function as both a promotional need (desire for more of the good) and a prevention need (desire for less of the bad). This duality is evident in common descriptions of individuals being motivated either by a “pull” or a “push.”^6^

The concept that leadership is essentially a process of guiding and facilitating followers toward individual and collective need fulfillment raises the question of *which* specific needs are involved. We argue that leadership is ultimately rooted in the fulfillment of a set of specific follower needs, in setting collective goals, providing a sense of safety, autonomy, fairness, ethics, success, recognition, authenticity, growth, absorption, caring, belonging, and purpose, and guiding followers to develop and achieve (McClelland, 1975; House and Aditya, 1997; House et al., 2004; Gagné and Deci, 2005; Deci and Ryan, 2008; Hogg et al., 2012). Accordingly, our analysis centers on coding leadership concepts and assessment items to the specific psychological needs that can be met to varying degrees through the decisions and actions of leadership.

## Results

Each of the 50 non-trait concepts and 267 items identified in the literature review could be classified according to one of the 12 emotional needs in our matrix. Table 2 displays the matrix with the distribution of concepts and assessment items.

- When examining leadership concepts and items, respectively, it is evident that there is a relatively balanced distribution across the three levels of striving, with roughly one-third^7^ of concepts and items falling into each of the Foundational (34.5% of concepts, 31.2% of items), Experiential (32.8, 35.7%) and Aspirational levels (32.8, 33.1%), suggesting that leadership concerns are spread equally across need levels.
- When the distribution of concepts and items, respectively, across the four life domains is examined,^8^ the distribution is nearly identical for the domains of the Self (20.2, 21.4%) and the Spiritual (21.0, 20.7%). The Material domain shows a roughly equivalent share of concepts (21.0%) but a larger share of assessment items (35.7%), with the heaviest coverage in the need for Autonomy (18.0%). The Social domain shows the opposite pattern, with roughly equal share of items (22.2%) as the Self and Spiritual domains, but greater concentration of concepts (37.8%) driven by a large share devoted to Inclusion concepts (19.3%).
- When the individual needs are inspected, we find relatively even coverage across eight of the 12 cells, or an expected rate of 8.3% per cell. The one need receiving scant attention is the need for Justice at only 1.7 percent of concepts and 1.1 percent of items, which may suggest that issues of fairness, equity, and justice, which have recently drawn significant management focus, may have been tacitly assumed to be sufficiently addressed in earlier decades when most of these instruments were created. This finding demonstrates the value of a comprehensive theoretical framework by clearly defining and measuring each discrete need. In sharp contrast to issues of Justice, a great deal of attention has shifted to questions of Ethics (14.3% of concepts and 14.7% of items), which was also found to hold a relatively large share in a recent analysis of the concepts of organizational culture (Pincus, 2024a), highlighting the conceptual linkage between leadership and culture.

**Table 2:** A unified model of human motivation (Pincus, 2022a) with distributions of leadership concepts and items.

Three modes of existence	Four life domains				Marginals
	Self	Material	Social	Spiritual	
Aspirational	Fulfilling potential & limitation	Success & failure	Recognition & scorn	Higher purpose & materialism	
% of leadership concepts	10.9%	6.7%	10.1%	5.0%	32.8%
% of leadership items	11.3%	9.0%	7.9%	4.9%	33.1%
Experiential	Authenticity & conformity	Immersion & stagnation	Caring & uncaring	Ethics & wrongdoing	
% of leadership concepts	5.0%	5.0%	8.4%	14.3%	32.8%
% of leadership items	4.5%	8.6%	7.9%	14.7%	35.7%
Foundational	Safety & insecurity	Autonomy & disempowerment	Inclusion & exclusion	Justice & injustice	
% of leadership concepts	4.2%	9.2%	19.3%	1.7%	34.5%
% of leadership items	5.6%	18.0%	6.4%	1.1%	31.2%
Marginals concepts	20.2%	21.0%	37.8%	21.0%	
Marginals items	21.4%	35.7%	22.2%	20.7%	

In the following section, we provide a brief description of the 12 emotional needs and corresponding leadership concepts.

### Motives of the self

#### Safety and insecurity

The need for safety is the most fundamental need in most models of motivation. When safety needs are salient, there are strivings for security, protection, and peace. Twelve major motivational systems list the need for safety as a fundamental need (Forbes, 2011; Pincus, 2022a). Approximately 4 % of leadership concepts and 6 % of items reflect psychological safety concerns. As noted above, items are split between those representing the leader’s perspective and those of the follower. Leader-focused items tend to address the leader’s ability to manage their own stress and anxiety. Follower-focused items reflect on their view of the leader’s anxiety-producing behaviors (e.g., invading my privacy, takes action only when problems get serious, delays responding to urgent questions, etc.). The concepts of *Laissez-faire Leadership, Transactional Leadership, Initiating Structure, Consideration,* and *Inequity* tend to be associated with the need for safety.

#### Authenticity and conformity

At the next, experiential level of the self-domain is the need and ability to bring one’s whole self to work (or whatever the social situation); this is the desire to view oneself as being different from others in a good way. Nine major motivational systems include the need for unique identity as a fundamental need (Forbes, 2011; Pincus, 2022a). Approximately 5 % of leadership concepts and assessment items reflect issues of personal authenticity. Leader-focused items that pertain to authenticity tend to reflect the leader’s willingness to offer their own ideas and innovations in unconventional or nontraditional, idiosyncratic ways. Follower-focused items reflect perceptions of the leader’s sensitivity to the follower’s individuality and unique needs. Authenticity needs are associated with leadership concepts of *Transformational Leadership*, *Unconventional Behavior,* and *Initiating Structure*.

#### Fulfilling potential and limitation

The culminating level of self-domain strivings is represented by the need for personal growth and development, to actualize or fulfill one’s potential. Eleven major motivational systems include personal growth or actualization as a fundamental need (Forbes, 2011; Pincus, 2022a). Approximately 11 % of leadership concepts and assessment items relate to fulfilling potential representing a relatively larger share. Leader-focused items that speak to the need for personal growth address the leader’s personal mastery of management skills (e.g., anticipating problems and planning for them; accurate perceptions, decisions, and predictions; keeping followers informed) and the leader’s active enablement of followers’ personal growth (e.g., I help others develop themselves). Follower-focused items relate to perceptions of a leader’s management skills and promotion of personal and career development. The need to fulfill personal potential is associated with leadership concepts of *Transformational Leadership, Strategic Vision and Articulation, Initiating Structure, Intellectual Stimulation*, *Individualized Consideration* and *Wisdom*.

### Motives of the material domain

#### Autonomy and disempowerment

The foundational need within the material domain is the striving for autonomy, to feel capable and permitted to take positive action. Seven major motivational systems feature this need, which goes by many names including autonomy, empowerment, self-efficacy, or self-determination (Forbes, 2011; Pincus, 2022a). The need for autonomy is associated with 9 % of leadership concepts and 18 percent of assessment items, the single largest share of items. Because the material domain is typically associated with the world of work and play, it is not surprising to see strong representation of these concepts. Leader-focused autonomy items pertain to the leader’s power and decision-making authority, decisiveness, persuasiveness, and empowerment of followers to make their own decisions. Follower-focused autonomy items pertain to perceptions of the leader’s delegation of responsibilities, assignments to tasks, sensitivity to constraints limiting follower autonomy, and openness to follower input and consensus. Leadership concepts associated with the need for autonomy include *Laissez-faire Leadership*, *Sensitivity to the Environment, Initiating Structure, Consideration,* and *Persuasive Mapping*.

#### Immersion and stagnation

At the next level of the material domain is the need for immersion, the striving to feel totally absorbed in the moment, often described as a state of *flow*. Thirteen major motivational systems include this motive (Forbes, 2011; Pincus, 2022a). The need for immersion is associated with 5 % of leadership concepts and 9 % of assessment items. Leader-focused immersion items relate to the leader’s emphasis on motivating productivity, efficiency, and hard work, as well as the provision of immediate performance-related feedback. Follower-focused items relate to their perceptions of the degree to which the leader is “tuned in” to the specifics of what is going on with the work itself. The need for immersion is associated with leadership concepts of *Initiating Structure, Consideration, Management-by-Exception* and *Wisdom*.

#### Success and failure

The material domain’s highest level of aspiration is the need for material success as the fruits of one’s labors. Seven major motivational systems include this motive (Forbes, 2011; Pincus, 2022a). The need for success is associated with 7 % of concepts and 9 % of items. Leader-focused success items focus on competitive, entrepreneurial spirit; setting clear goals to which rewards are tied; provision of holistic performance feedback; and the ability to handle failures. Follower-focused items relate to perceptions of the leader’s embracing of goals, clarifying goal-related expectations, and being overly focused on the follower’s mistakes or failures. Leadership concepts over-represented in the need for success include *Transactional Leadership*, *Transformational Leadership, Contingent Reward*, *Initiating Structure*, and *Strategic Vision and Articulation*.

### Motives of the social domain

#### Inclusion and exclusion

The most basic, foundational level of the social domain is the need for social inclusion and belonging that is the gateway to close relationships and social admiration. Nine major motivational systems include this need (Forbes, 2011; Pincus, 2022a). The need for inclusion is associated with the single largest share of leadership concepts, 19 percent, yet only 6 % of items. Leader-focused items addressing the need for inclusion tend to focus on the leader’s self-perceived sociability, conflict resolution skills, and building and maintaining group cohesion. Follower-focused items reflect opinions of the leader’s fostering a sense of community and fellowship. Leadership concepts associated with the need for inclusion include *Consideration, Organizational Stewardship, Altruistic Calling, Emotional Healing,* and *Self-Awareness*.

#### Caring and uncaring

The next level of the social domain is the need for mutually giving, intimate relationships. Eight major theories of motivation include the need for attachment, intimacy, or nurturance (Forbes, 2011; Pincus, 2022a). The need for caring is associated with 8 % of leadership concepts and items. Leader-focused caring items pertain to sincere concern for the welfare of followers, being approachable and sensitive to concerns, and putting the needs of followers first. Follower-focused items relate to perceptions of the leader’s positive, empathic behaviors (e.g., shows sensitivity, listens, etc.) and uncaring, negative behaviors (e.g., rude, ridicules, calls me stupid, etc.). The need for caring is associated with leadership concepts of *Hostility (*reversed*)*, *Consideration, Emotional Healing, Concern for Stakeholders, Loyalty, Affect,* and *Sensitivity to Member Needs*.

#### Recognition and scorn

The highest level of the social domain is the striving for esteem, respect, validation, affirmation, and admiration. Eight major motivational systems include this need (Forbes, 2011; Pincus, 2022a). The need for recognition is associated with 10 % of leadership concepts and 8% of items. Leader-focused items relate to the leader’s self-perceived role in instilling earned respect and representing and publicizing the team to others in the organization. Follower-focused items relate to the degree to which followers respect the leader, as well as an emphasis on negative, scorn-inducing behaviors (e.g., steals credit, blames me to save themselves from embarrassment, lowers my esteem in the group). Leadership concepts that address the need for recognition include *Transformational Leadership, Initiating Structure, Sensitivity to Member Needs, Contingent Reward,* and *Idealized Influence*.

### Motives of the spiritual domain

#### Justice and injustice

The spiritual domain represents the antipode of the material domain. If the material domain is fundamentally about visible and tangible reality, the spiritual domain concerns the world of invisible ideals and principles. The foundational level of the spiritual domain is the need for fairness and justice, the idea that ultimately good is rewarded and bad is punished. At least five major motivational systems include the justice motive, especially those addressing moral development [e.g., those of Kohlberg (1973), Lerner (2003), Bloom (2014), Haidt (2008), Greene (2014); reviewed in Pincus (2022a)]. Colquitt et al. (2001) have reviewed the extensive literature on organizational justice research, which has emerged as a separate subdiscipline. In the light of a host of news reports concerning social justice, the need for justice receives by far the fewest mentions in the leadership literature, only 2 % of concepts and 1 % of items. Of the three justice-related items, two are leader-focused items (e.g., *I treat all group members as my equals*). The single follower-focused item is *Makes fair and balanced decisions*. Leadership concepts associated with the need for justice include *Fairness* and *Consideration*.

#### Ethics and wrongdoing

The next level of the spiritual domain is the need for ethical conduct, striving for behavior that is consistent with moral values, which are built on a platform of basic justice. At least five major motivational systems include this need and tend to be those focused on moral development [e.g., those of Kohlberg (1973), Batson et al. (2005), Staub (2005), Haidt (2008), and Kant and Paton (1964); reviewed in Pincus (2022a)]. In sharp contrast to the need for justice, the need for ethics is well-populated by leadership items and concepts, representing approximately 14 percent of both concepts and items. Leader-focused items pertain to the leader’s self-perceived ethical, moral conduct and requiring the same from employees, limiting self-interest, and acting with honesty and transparency. Follower-focused items focus on perceptions of the leader’s adherence to ethical standards, delivering on promises, and encouraging followers to “give back” to the community. Leadership concepts relating to the need for ethics include *Transformational Leadership, Personal Risk*, *Contribution, Relational Transparency*, *Moral Person, Moral Manager, Organizational Stewardship, Internalized Moral Perspective*, *Initiating Structure,* and *Altruistic Calling*.

#### Higher purpose and materialism

The apex of the spiritual domain is represented by the highest and noblest striving, the need to serve a higher calling. The need for a transcendent higher purpose is featured in at least five major motivational-developmental systems, including the contributions of, Kant and Paton (1964), James (1890), Frankl (1985), Maslow (1970), and Kohlberg (1973); reviewed in Pincus (2022a). In terms of the amount of representation, this need falls in between the need for justice and the need for ethics at 5 % of concepts and items. Leader-focused items pertain to helping followers find meaning, mission, and purpose in their work, sincerely believing that there is a higher purpose to their own work and providing an inspirational vision to followers. Follower-focused items pertain to perceptions of the leader’s sincerity of conviction about making a positive difference in the world and in the future. Leadership concepts pertaining to the need for purpose include *Inspirational Motivation*, *Transformational Leadership*, *Strategic Vision and Articulation*, and *Organizational Stewardship*.

## Discussion

The major finding of the analysis is that non-trait leadership concepts readily find homes in discrete emotional needs, and that the distribution of these needs across levels of aspiration and life domains is relatively even. This finding strongly supports the contention that a structured framework of follower needs can provide the meta-theory sought by the leadership field. We argue that leadership constructs are best described as points of intersection between the psychological needs of individual followers and the decisions, actions, and resources championed by leadership, resulting in variable levels of fulfillment across the landscape of needs. The core concept here is *motivation*. Motivations represent pent up energies caused by unmet needs, which direct organisms to seek fulfilled, balanced, homeostatic states. It seems that the strong degree of fit between leadership concepts and our framework of follower needs is not a coincidence.

**Figure 2:**
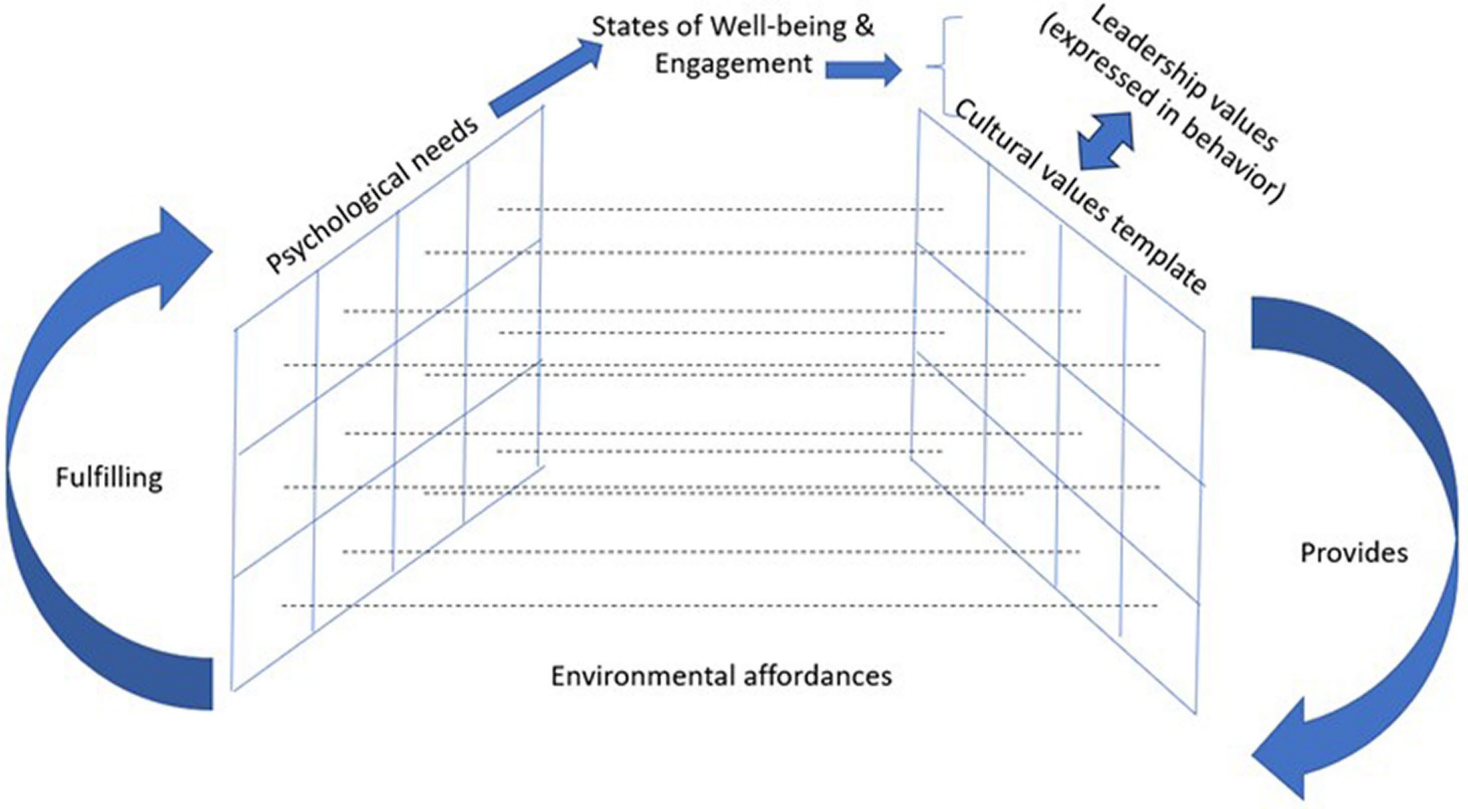
Leadership and culture interact to provide environmental affordances that fulfill psychological needs resulting in relative states of well-being & engagement.

Applying the needs framework provides us with an opening to integrate a wide range of fundamental organizational concepts: employee engagement (Pincus, 2022b), employee well-being (Pincus, 2023a), organizational culture (Pincus, 2024a), organizational values (Pincus, 2024b), and the role of leadership in influencing all of these. Employee *subjective well-being* is the product of the comparison of environmental affordances against psychological needs. To the extent that needs are met, a *healthy* culture will be inferred; to the extent that needs go unmet, the culture will be considered *toxic*. Those working in healthy cultures, where psychological needs are fulfilled (producing states of *well-being*) enjoy their work and can be viewed as highly *engaged*. Those suffering under toxic cultures, where needs go unmet (producing states of *ill-being*), dislike their work, and can be viewed as actively *disengaged*. In this model, leadership plays an important, outsized role in determining the parameters of organizational culture. It is leadership, ultimately, that prioritizes environmental resources that bespeak the organization’s *values*. Such values represent the level of priority that the organization places on satisfying each particular need. For example, some organizations value ethics and purpose at the expense of maximizing profits, whereas others value excellence and achievement above all other considerations; in both cases, employee needs are prioritized accordingly. To this end, we propose a theoretical hierarchy for conceptualizing the dimensions of leadership within a larger context of culture, values, well-being, and employee engagement (Figure 2).

Leadership’s actions, which reinforce values, interact dynamically with the existing organizational culture, forming an iterative process that evolves with each proclamation and initiative. Leadership behaviors exert a tangible and influential force that shapes the organizational culture and impacts how the organization is perceived internally and externally. They significantly contribute to judgments regarding whether the organization is a desirable place to work and whether it serves as a force for good, evil, or somewhere in between. These associations form essential components of the organization’s reputation, directly affecting its ability to meet the needs of its employees, customers, and society at large. It is within the realm of fulfilling these twelve emotional needs that the true essence of leadership unfolds.

### Implications for theory

The challenge of precisely defining and operationalizing the concept of leadership has been extensively acknowledged in the literature (Yukl et al., 2002; Le et al., 2010; Yukl, 2010, 2012; DeRue et al., 2011; Shaffer et al., 2016; Antonakis, 2017). According to Schneider et al. (2013), one reason for the lack of clarity in articulating this construct may be the flexibility afforded to practitioners by a loose definition, allowing consultants to manipulate and adapt the concept to their liking. However, we contend that the costs associated with unclear definitions far outweigh any benefits practitioners may perceive from operating without boundaries.

The absence of a unifying theoretical foundation has led to the proliferation of concepts in the field of leadership, as evidenced by the extensive number of concepts (50) and assessment items (267) identified in the literature review, with little consistency across different models. This conceptual confusion creates a metaphorical “white-out” condition, making it challenging to navigate through the overlapping concepts and indicating a failure to address the fundamental nature of leadership.

It is important to note that the needs-based framework is able to match the content of leadership concepts in a far more parsimonious manner than traditional leadership categories, which tend to be very “big tents” that hold many distinct ideas. For example, the leadership concept of Transformational Leadership is associated with content related to needs for authenticity, potential, success, recognition, ethics, and purpose. Another example is the leadership concept of Consideration, which is associated with the needs for safety, potential, authenticity, immersion, inclusion, caring, and justice. The most significant contribution of the application of the matrix, in our opinion, is its ability to clean up and organize the seemingly endless parade of concepts. It is our hope that we have provided a comprehensive structured framework for thinking about leadership that may slow the pace of concept proliferation as new constructs can be categorized among similar constructs in shared cells of the framework.

A secondary advantage accruing from the application of the matrix is the ability to judge the degree that each of the twelve needs are covered in theory (i.e., in terms of dimensions) and in measurement (i.e., in terms of assessment items). As suggested, surprisingly little attention has been historically paid to the need for justice. Important underrepresented themes can now be easily identified and added to future theory and measurement development.

The emotional needs framework further postulates that every need can operate as either a promotion or prevention need. Theory development has tended to stumble over this distinction, with certain needs well-covered by negatives (i.e., conflict as the opposite of safety; lacking authority as the opposite of autonomy; hostility as the opposite of caring; etc.), while others are assessed only in their positive expression. Because they are experienced differently, and demand different treatments, it is our hope that future theory and measurement will formally distinguish between promotion and prevention needs.

Our aim is to contribute to the development of theory by establishing a comprehensive framework for leadership action that encompasses all higher-order human needs. Our model of emotional needs can be depicted as a pyramid, with the four life domains represented on its four faces. These domains are organized as pairs of opposites: self-social and material-spiritual. Using a distance metaphor, our model predicts stronger associations among adjacent domains (e.g., self-material-social) and weaker associations for domains that are antipodal (self-social, material-spiritual). This proposition has garnered significant theoretical and empirical support from studies conducted by Kohlberg and Power (1981), Mahoney et al. (2005), Coelho et al. (2019), Bilsky et al. (2011), Oishi and Diener (2009), and Pincus (2023b).

A key objective for future research is to elucidate the interplay between emotional needs and the varying degrees to which leadership fulfills them, thereby promoting significant outcomes such as enhanced perceptions of a healthy culture, improved employee subjective well-being, and increased engagement. Our model proposes that this progression entails a process of de-centering, wherein individuals shift their focus from themselves to the external world, then to the social realm, and ultimately to the realm of principles. As needs are fulfilled, further advancement involves transcending the individual definitions of each need, as all twelve needs gradually merge together. For instance, what fosters a sense of achievement also serves as an example of ethical behavior, and what instills a sense of security also promotes justice for others. Likewise, experiences of authenticity align with respect, and so forth. This fusion of needs signifies an integrated and interconnected framework, wherein the fulfillment of one need contributes to the fulfillment of others, resulting in a holistic progression of leadership activities and effects.

### Implications for methods

Similar to the challenges faced in measuring subjective well-being, employee engagement, and organizational culture, the field of leadership research has encountered difficulties in developing measurement approaches that overcome the limitations associated with written statements and numerical rating scales. The sensitive nature of employee ratings on leadership introduces a significant challenge known as the “fake-ability” of responses. This issue is particularly relevant for employees who are hesitant to speak the truth of their experience to managers who may react negatively to criticism. Ideally, approaches to measuring leadership practices and effects should minimize the potential for response filtering, control, and faking.

We contend that a fundamental shift in measuring leadership concepts is necessary. Recognizing that the impact of leadership is ultimately experienced through the fulfillment of needs, an inherently motivational-emotional process, we argue that relying solely on numerically rated verbal statements is intrinsically flawed. Such approaches rely on rational and analytical thinking, rather than capturing emotions or feelings. Fortunately, there are alternative approaches, collectively known as “System 1” approaches, designed to bypass cognitive filters and directly measure motivational-emotional processes. System 1 techniques encompass various methods, including brain imaging techniques like fMRI, MEG, NIRS, and EEG, psychophysiological measures such as facial coding, galvanic skin response, eye tracking, cardiac functioning, and respiration, as well as scalable indirect measures of motivational-emotional meaning like time-constrained image-based elicitation (Pincus, 2023b). Given that the effects and conditions of leadership are primarily experienced through emotional channels, it is imperative to employ measurement methods that align with its affective nature.

### Implications for practice

The absence of a meta-theoretical framework has had a noticeable impact on the advancement of leadership theory and measurement. Without a unified framework to organize the multitude of items and dimensions proposed, progress has been impeded. In response, we aim to provide a solution by offering a comprehensive and integrated framework that consolidates these various elements. Our intention is for this framework to benefit not only theorists seeking theoretical clarity but also practitioners who require a structured approach to describe their frameworks and measures to clients. We firmly believe that our model offers significant advantages in terms of its structure. By categorizing needs according to life domains and levels of striving, it establishes a hierarchical order that provides a clear understanding. This structure not only indicates which need fulfillments contribute to progress within each domain but also identifies the ones that naturally co-occur and those that may potentially oppose one another. Thanks to these structural assumptions, the model can generate testable hypotheses, facilitating the comprehension of intervention impacts on sets of needs. Adopting a holistic meta-theory rooted in first principles can greatly simplify the work of theorists, researchers, and practitioners. It establishes a shared framework that ensures all fundamental concepts are given equal representation, streamlining the overall process.

### Limitations and recommendations

The primary limitation of this study pertains to the positivistic orientation of the analysis which focused on traditional leader-centric conceptualizations of leadership. There are alternative theoretical perspectives, notably those stemming from the Social Identity tradition, which tend to emphasize the importance of the interplay between individual psychology and the social environment in co-creating social dynamics.^9^ We find no discrepancy between our categorical method for understanding the human needs addressed (or unaddressed) by leadership and the alternative tradition that examines the relational dynamics of leadership. No matter the mechanisms through which leadership values are established, sustained, or transformed, the overarching human needs they can fulfill remain constant. The dynamic interactions that shape leadership invariably involve compromises between personal needs (for example, balancing the need for security with the risks necessary for growth), as well as between the needs of different individuals (such as an employee’s desire for purpose versus a manager’s obligation to deliver profits), and between personal needs and those of the organization (like balancing an individual’s need for independence and authenticity against the organization’s requirements for uniformity and focused objectives).

A second limitation involves our aim to associate leadership behaviors and styles with the needs they address. Clearly, needs cannot be directly matched to leadership behaviors and styles in a straightforward one-to-one relationship because both are part of many-to-many relationships. In other words, a specific leadership behavior or style may satisfy various needs, and conversely, a specific need may be satisfied by various leadership behaviors and styles. Despite the complexity of creating a matrix that links needs to leadership behaviors and styles, we believe such a project is feasible and beneficial, as it would align individual needs with appropriate leadership strategies. A critical aspect to consider in establishing these connections is that leadership behaviors and styles are often formulated based on recognized needs. The development of leadership strategies within an organization should occur through a collaborative process between employees and management, where needs are identified and addressed as a priority. This approach should be standard practice, yet, to date, there is little evidence of systematic evaluation of emotional needs in organizations.

Our recommendations arise directly from this observation. Organizations often assume they understand the needs of individuals, yet the typical organization can show no evidence of such understanding. Leadership practices have become critically important due to the failure of organizations to accurately comprehend and address the needs of their employees and customers. This shortfall is highlighted by recurring leadership failures and scandals. We urge stakeholders to adopt our framework as a basis for assessing the individual needs within organizations and for identifying the necessary leadership practices to effectively meet these needs.

## Conclusion

In response to the expanding dimensions and concepts found in leadership literature, this paper addresses the pressing demand for integration. To meet this need, a meta-theory is presented, capable of encompassing the ever-increasing assortment of leadership constructs. The proposed meta-theory is rooted in twelve fundamental human needs, providing a solid theoretical foundation. Given the allocation of substantial resources toward resolving critical leadership failures, it is imperative to establish a coherent and comprehensive framework. Without such a framework, the measurement methods and interventions employed run the significant risk of being inconsistent and unreliable.

## Data availability statement

The datasets presented in this study can be found in online repositories. The names of the repository/repositories and accession number(s) can be found in the article/Supplementary material.

## Author contributions

JP: Conceptualization, Formal analysis, Writing – original draft, Writing – review & editing.

## Footnotes

^1^According to the latest Gallup global data, there are roughly the same percentage of “engaged” and “actively disengaged” employees (about 20 percent each of the global workforce). The remaining 60 percent are merely “disengaged.” (Gallup Organization, 2023).

^2^With the addition of *Laissez-Faire* leadership, these subtypes have been summarized as the Full-Range Leadership Model (Antonakis and House, 2013).

^3^Search results ranged from 247,000 results for *altruistic leadership* to over 6 million results each for *effective*, *team*, and *shared* leadership.

^4^See discussion of social identity (or relational dynamic) perspectives in the Discussion/Limitations section below.

^5^According to Aristotle (1933), existence can be categorized into three states: potentiality, potentiality-as-such (the action that brings potential closer to reality), and actuality (the end result). To illustrate this concept, he referred to the construction of a house. The materials possess the potential to be used for building a house or for other purposes, representing their state of potentiality, which Aristotle termed “the buildable.” The process of building, however, converts the materials into their intended form, progressing them from potentiality towards actualization, which Aristotle referred to as potentiality-as-such. Finally, when the construction is completed, the materials have reached a state of actuality.

^6^Individuals can find motivation in both positive aspirations and the avoidance of negative frustrations related to the same motivation. They may be motivated by one or the other, by both or by neither. Since these forces operate in a complementary way, we have not made distinct predictions regarding the functioning of positive and negative needs.

^7^The expected rate if randomly distributed would be 33.3% in each of the three levels.

^8^The expected rate if randomly distributed would be 25.0% in each of the four domains.

^9^The Social Identity Theory of Leadership suggests that leadership effectiveness depends largely on the leader’s ability to embody and promote the group’s identity and values. This theory, which tends to be associated with the work of Hogg (Hogg et al., 2012), builds on the broader Social Identity Theory, which explores how people’s self-concepts are shaped by their membership in social groups. According to this leadership theory, leaders are more likely to be effective and influential if they are perceived as prototypical of the group—that is, embodying the characteristics, values, and norms that define the group’s identity. Leaders who are seen as one of the team can enhance group cohesion and motivate members by emphasizing shared identity and common goals, ultimately influencing the meaning of group membership (see Haslam and Platow, 2001; Haslam et al., 2010, 2017).
