# Corrigendum: Leadership as a determinant of need fulfillment: implications for meta-theory, methods, and practice

**DOI:** 10.3389/fpsyg.2024.1462854

**Published:** 2024-08-07

**Authors:** J. David Pincus

**Affiliations:** Leading Indicator Systems, Boston, MA, United States

**Keywords:** leadership, transformational leadership, servant leadership, sustainable leadership, toxic leadership, organizational culture, employee engagement, employee well-being

In the published article, there was an error in the affiliation. Instead of “Employee Benefit Research Institute, Washington, DC, United States”, it should be “Leading Indicator Systems, Boston, MA, United States.”

In the published article, there was an error. Due to the change of affiliation, the Conflict of Interest statement is incorrect.

A correction has been made to **Conflict of Interest**, this sentence previously stated:

The author declares that the research was conducted in the absence of any commercial or financial relationships that could be construed as a potential conflict of interest.

The corrected sentence appears below:

JP was employed by Leading Indicator Systems, Boston.

The author apologizes for these errors and states that this does not change the scientific conclusions of the article in any way. The original article has been updated.

