# Supplementary material for: Leadership as a determinant of need fulfillment: implications for meta-theory, methods, and practice
Source: Front Psychol. 2024 Jun 25;15:1427072. doi: 10.3389/fpsyg.2024.1427072 (PMC11337100; doi:10.3389/fpsyg.2024.1427072)
Supplement: Supplementary file 1 [file Table_1.docx]

Supplementary Material

Supplementary Table 1. Leadership concepts and assessment items by emotional needs.

| Need category | Assessment item (* = negatively worded) | Concept | Source |
| --- | --- | --- | --- |
| Safety (A1) | Avoids getting involved when important issues arise* | Laissez-faire Leadership | Avolio, et al. (1999) |
| Safety (A1) | Is absent when needed* | Laissez-faire Leadership | Avolio, et al. (1999) |
| Safety (A1) | Delays responding to urgent questions* | Laissez-faire Leadership | Avolio, et al. (1999) |
| Safety (A1) | Does not interfere until the problem is serious* | Transactional leadership | Avolio, et al. (1999) |
| Safety (A1) | Believes in not making changes unless necessary* | Transactional leadership | Avolio, et al. (1999) |
| Safety (A1) | Takes action only when problem become serious* | Transactional leadership | Avolio, et al. (1999) |
| Safety (A1) | I become anxious when I cannot find out what is coming next* | Initiating Structure | Halpin & Stogdill (1962) |
| Safety (A1) | I let some members take advantage of me* | Consideration | Halpin & Stogdill (1962) |
| Safety (A1) | I become anxious when waiting for new developments* | Consideration | Halpin & Stogdill (1962) |
| Safety (A1) | l am hesitant about taking initiative in the group* | Initiating Structure | Halpin & Stogdill (1962) |
| Safety (A1) | I get swamped by details* | Initiating Structure | Halpin & Stogdill (1962) |
| Safety (A1) | I can wait just so long, then blow up* | Consideration | Halpin & Stogdill (1962) |
| Safety (A1) | I remain calm when uncertain about coming events | Initiating Structure | Halpin & Stogdill (1962) |
| Safety (A1) | I get confused when too many demands are made of me* | Initiating Structure | Halpin & Stogdill (1962) |
| Safety (A1) | My boss invades my privacy* | Inequity | Tepper (2000) |
| Authenticity (A2) | Treats me as individual rather than member of group | Transformational leadership | Avolio, et al. (1999) |
| Authenticity (A2) | Considers me as having different needs/ abilities / aspiration | Transformational leadership | Avolio, et al. (1999) |
| Authenticity (A2) | Engages in unconventional behavior in order to achieve organizational goals. | Unconventional Behavior | Conger, Kanungo, & Menon (2000) |
| Authenticity (A2) | Use nontraditional means to achieve organizational goals. | Unconventional Behavior | Conger, Kanungo, & Menon (2000) |
| Authenticity (A2) | Often exhibits very unique behavior that surprises other members of the organization. | Unconventional Behavior | Conger, Kanungo, & Menon (2000) |
| Authenticity (A2) | I try out my ideas in the group | Initiating Structure | Halpin & Stogdill (1962) |
| Authenticity (A2) | I make my attitudes clear to the group | Initiating Structure | Halpin & Stogdill (1962) |
| Authenticity (A2) | I am willing to make changes | Consideration | Halpin & Stogdill (1962) |
| Authenticity (A2) | I persuade others that my ideas are to their advantage | Initiating Structure | Halpin & Stogdill (1962) |
| Authenticity (A2) | I worry about the outcome of any new procedure* | Initiating Structure | Halpin & Stogdill (1962) |
| Authenticity (A2) | My department manager is sensitive to department employees’ responsibilities outside the workplace. | Emotional Healing | Ehrhart (2004) |
| Authenticity (A2) | As a leader I encourage everyone to speak their mind | Relational transparency | Luthans & Avolio (2003) |
| Potential (A3) | I enable others to think about old problems in new ways. | Intellectual Stimulation | Bass & Avolio (1992) |
| Potential (A3) | I help others develop themselves. | Individualized Consideration | Bass & Avolio (1992) |
| Potential (A3) | I provide others with new ways of looking at puzzling things. | Intellectual Stimulation | Bass & Avolio (1992) |
| Potential (A3) | I get others to rethink ideas that they had never questioned before. | Intellectual Stimulation | Bass & Avolio (1992) |
| Potential (A3) | Seeking different perspective in problem solving | Transformational leadership | Avolio, et al. (1999) |
| Potential (A3) | Allows me look at problems different angles | Transformational leadership | Avolio, et al. (1999) |
| Potential (A3) | Suggests new ways to completing my work | Transformational leadership | Avolio, et al. (1999) |
| Potential (A3) | Spends time on training and caching | Transformational leadership | Avolio, et al. (1999) |
| Potential (A3) | Helps me to develop my strength. | Transformational leadership | Avolio, et al. (1999) |
| Potential (A3) | Consistently generates new ideas for the future of the organization. | Strategic Vision and Articulation | Conger, Kanungo, & Menon (2000) |
| Potential (A3) | Has vision; often brings up ideas about possibilities for the future. | Strategic Vision and Articulation | Conger, Kanungo, & Menon (2000) |
| Potential (A3) | How well does your leader (follower) recognize your potential? | Professional Respect | Graen & Uhl-Bien (1995) |
| Potential (A3) | I fail to take necessary actions* | Initiating Structure | Halpin & Stogdill (1962) |
| Potential (A3) | I wait patiently for the results of a decision | Consideration | Halpin & Stogdill (1962) |
| Potential (A3) | I am able to predict what is coming next | Initiating Structure | Halpin & Stogdill (1962) |
| Potential (A3) | I accept delays without becoming upset | Consideration | Halpin & Stogdill (1962) |
| Potential (A3) | I give advance notice of changes | Consideration | Halpin & Stogdill (1962) |
| Potential (A3) | Things usually turn out as I predict | Initiating Structure | Halpin & Stogdill (1962) |
| Potential (A3) | I am able to tolerate postponement and uncertainty | Consideration | Halpin & Stogdill (1962) |
| Potential (A3) | I am accurate in predicting the trend of events | Initiating Structure | Halpin & Stogdill (1962) |
| Potential (A3) | I get things all tangled up* | Initiating Structure | Halpin & Stogdill (1962) |
| Potential (A3) | I am able to delay action until the proper time occurs | Consideration | Halpin & Stogdill (1962) |
| Potential (A3) | I anticipate problems and plans for them | Initiating Structure | Halpin & Stogdill (1962) |
| Potential (A3) | I make accurate decisions | Initiating Structure | Halpin & Stogdill (1962) |
| Potential (A3) | My department manager makes the personal development of department employees a priority. | Altruistic Calling/Commitment to the Growth of People | Ehrhart (2004) |
| Potential (A3) | My department manager displays wide-ranging knowledge and interests in finding solutions to work problems. | Wisdom | Ehrhart (2004) |
| Potential (A3) | My department manager works hard at finding ways to help others be the best they can be. | Commitment to the Growth of People | Ehrhart (2004) |
| Potential (A3) | My boss tells me I'm incompetent* | Undermining | Tepper (2000) |
| Potential (A3) | As a leader I accurately describe how others view my capabilities | Self-awareness | Luthans & Avolio (2003) |
| Potential (A3) | Skills and abilities (including social skills) | Mediators | Judge, et al. (2004) |
| Autonomy (B1) | I can reduce a madhouse to system and order | Initiating Structure | Halpin & Stogdill (1962) |
| Autonomy (B1) | I let others take away my leadership in the group* | Initiating Structure | Halpin & Stogdill (1962) |
| Autonomy (B1) | I back down when I ought to stand firm* | Initiating Structure | Halpin & Stogdill (1962) |
| Autonomy (B1) | I express with a few simple words what we could and should do. | Inspirational Motivation | Bass & Avolio (1992) |
| Autonomy (B1) | I am content to let others continue working in the same way as always. | Laissez-faire Leadership | Bass & Avolio (1992) |
| Autonomy (B1) | Whatever others want to do is okay with me. | Laissez-faire Leadership | Bass & Avolio (1992) |
| Autonomy (B1) | I tell others the standards they have to know to carry out their work. | Management-by-exception | Bass & Avolio (1992) |
| Autonomy (B1) | I ask no more of others than what is absolutely essential. | Laissez-faire Leadership | Bass & Avolio (1992) |
| Autonomy (B1) | Avoids making decisions* | Laissez-faire Leadership | Avolio, et al. (1999) |
| Autonomy (B1) | Discusses with specific terms who is responsible for achieving performance targets | Transactional leadership | Avolio, et al. (1999) |
| Autonomy (B1) | Displays sense of power and confidence | Transformational leadership | Avolio, et al. (1999) |
| Autonomy (B1) | Readily recognizes constraints in the physical environment. | Sensitivity to the Environment | Conger, Kanungo, & Menon (2000) |
| Autonomy (B1) | Readily recognizes constraints in the organization’s social and cultural environment. | Sensitivity to the Environment | Conger, Kanungo, & Menon (2000) |
| Autonomy (B1) | Recognizes the abilities and skills of other members of the organization. | Sensitivity to the Environment | Conger, Kanungo, & Menon (2000) |
| Autonomy (B1) | Recognizes the limitations of other members of the organization. | Sensitivity to the Environment | Conger, Kanungo, & Menon (2000) |
| Autonomy (B1) | How well does your leader (follower) understand your job problems and needs? | Professional Respect | Graen & Uhl-Bien (1995) |
| Autonomy (B1) | My arguments are convincing | Initiating Structure | Halpin & Stogdill (1962) |
| Autonomy (B1) | I permit the members to use their own judgment in solving problems | Consideration | Halpin & Stogdill (1962) |
| Autonomy (B1) | I argue persuasively for my point of view | Initiating Structure | Halpin & Stogdill (1962) |
| Autonomy (B1) | I encourage initiative in the group members | Consideration | Halpin & Stogdill (1962) |
| Autonomy (B1) | I put suggestions made by the group into operation | Consideration | Halpin & Stogdill (1962) |
| Autonomy (B1) | I am a very persuasive talker | Initiating Structure | Halpin & Stogdill (1962) |
| Autonomy (B1) | I let the members do their work the way they think best | Consideration | Halpin & Stogdill (1962) |
| Autonomy (B1) | I let group members know what is expected of them | Initiating Structure | Halpin & Stogdill (1962) |
| Autonomy (B1) | I am very skillful in an argument | Initiating Structure | Halpin & Stogdill (1962) |
| Autonomy (B1) | I decide what shalI be done and how it shall be done | Initiating Structure | Halpin & Stogdill (1962) |
| Autonomy (B1) | I assign a task, then lets the members handle it | Initiating Structure | Halpin & Stogdill (1962) |
| Autonomy (B1) | I allow the members complete freedom in their work | Consideration | Halpin & Stogdill (1962) |
| Autonomy (B1) | I am not a very convincing talker* | Initiating Structure | Halpin & Stogdill (1962) |
| Autonomy (B1) | I assign group members to particular tasks | Initiating Structure | Halpin & Stogdill (1962) |
| Autonomy (B1) | I turn the members loose on a job, and lets them go to it | Consideration | Halpin & Stogdill (1962) |
| Autonomy (B1) | I am reluctant to allow the members any freedom of action* | Consideration | Halpin & Stogdill (1962) |
| Autonomy (B1) | I let some members have authority that I should keep* | Initiating Structure | Halpin & Stogdill (1962) |
| Autonomy (B1) | My word carries weight with his superiors | Consideration | Halpin & Stogdill (1962) |
| Autonomy (B1) | I schedule the work to be done | Initiating Structure | Halpin & Stogdill (1962) |
| Autonomy (B1) | I allow the group a high degree of initiative | Consideration | Halpin & Stogdill (1962) |
| Autonomy (B1) | I take full charge when emergencies arise | Initiating Structure | Halpin & Stogdill (1962) |
| Autonomy (B1) | I maintain definite standards of performance | Initiating Structure | Halpin & Stogdill (1962) |
| Autonomy (B1) | I trust the members to exercise good judgment | Consideration | Halpin & Stogdill (1962) |
| Autonomy (B1) | I overcome attempts made to challenge my leadership | Initiating Structure | Halpin & Stogdill (1962) |
| Autonomy (B1) | I act without consulting the group* | Consideration | Halpin & Stogdill (1962) |
| Autonomy (B1) | My department manager’s decisions are influenced by departmental employees’ input. | Persuasive Mapping | Ehrhart (2004) |
| Autonomy (B1) | My department manager tried to reach consensus among department employees on important decisions. | Persuasive Mapping | Ehrhart (2004) |
| Autonomy (B1) | This person offers compelling reasons to get me to do things | Persuasive mapping | Barbuto & Wheeler (2006) |
| Autonomy (B1) | This person encourages me to dream "big dreams" about the organization | Persuasive mapping | Barbuto & Wheeler (2006) |
| Autonomy (B1) | This person is very persuasive | Persuasive mapping | Barbuto & Wheeler (2006) |
| Autonomy (B1) | This person is very good at convincing me to do things | Persuasive mapping | Barbuto & Wheeler (2006) |
| Autonomy (B1) | This person is gifted when it comes to persuading me | Persuasive mapping | Barbuto & Wheeler (2006) |
| Immersion (B2) | I let others know how I think they are doing. | Individualized Consideration | Bass & Avolio (1992) |
| Immersion (B2) | As long as things are working, I do not try to change anything. | Management-by-exception | Bass & Avolio (1992) |
| Immersion (B2) | Exciting public speaker | Strategic Vision and Articulation | Conger, Kanungo, & Menon (2000) |
| Immersion (B2) | I encourage the use of uniform procedures | Initiating Structure | Halpin & Stogdill (1962) |
| Immersion (B2) | I needle members for greater effort* | Consideration | Halpin & Stogdill (1962) |
| Immersion (B2) | I make pep talks to stimulate the group | Consideration | Halpin & Stogdill (1962) |
| Immersion (B2) | I keep the work moving at a rapid pace | Initiating Structure | Halpin & Stogdill (1962) |
| Immersion (B2) | I push for increased production | Initiating Structure | Halpin & Stogdill (1962) |
| Immersion (B2) | I handle complex problems efficiently | Initiating Structure | Halpin & Stogdill (1962) |
| Immersion (B2) | I ask the members to work harder | Initiating Structure | Halpin & Stogdill (1962) |
| Immersion (B2) | I permit the members to take it easy in their work* | Initiating Structure | Halpin & Stogdill (1962) |
| Immersion (B2) | I see to it that the work of the group is coordinated | Initiating Structure | Halpin & Stogdill (1962) |
| Immersion (B2) | I drive hard when there is a job to be done | Initiating Structure | Halpin & Stogdill (1962) |
| Immersion (B2) | I encourage overtime work | Initiating Structure | Halpin & Stogdill (1962) |
| Immersion (B2) | I can inspire enthusiasm for a project | Consideration | Halpin & Stogdill (1962) |
| Immersion (B2) | I permit the group to set its own pace | Consideration | Halpin & Stogdill (1962) |
| Immersion (B2) | I keep the group working up to capacity | Initiating Structure | Halpin & Stogdill (1962) |
| Immersion (B2) | My department manager balances concern for day-to-day details with projections for the future. | Wisdom | Ehrhart (2004) |
| Immersion (B2) | This person seems alert to what's happening | Wisdom | Barbuto & Wheeler (2006) |
| Immersion (B2) | This person is good at anticipating the consequences of decisions | Wisdom | Barbuto & Wheeler (2006) |
| Immersion (B2) | This person has great awareness of what's going on | Wisdom | Barbuto & Wheeler (2006) |
| Immersion (B2) | This person seems in touch with what's happening | Wisdom | Barbuto & Wheeler (2006) |
| Immersion (B2) | This person seems to know what is going to happen | Wisdom | Barbuto & Wheeler (2006) |
| Success (B3) | I tell others what to do if they want to be rewarded for their work. | Contingent Reward | Bass & Avolio (1992) |
| Success (B3) | I am satisfied when others meet agreed-upon standards. | Management-by-exception | Bass & Avolio (1992) |
| Success (B3) | I call attention to what others can get for what they accomplish. | Contingent Reward | Bass & Avolio (1992) |
| Success (B3) | Provides with assistants an exchange for my effort | Transactional leadership | Avolio, et al. (1999) |
| Success (B3) | Clarifies my expectation when meeting perform expectation goal | Transactional leadership | Avolio, et al. (1999) |
| Success (B3) | Expresses satisfaction when meeting performance | Transactional leadership | Avolio, et al. (1999) |
| Success (B3) | Focuses attention on irregularities /mistake deviation from standards* | Transactional leadership | Avolio, et al. (1999) |
| Success (B3) | Gives all attention in dealing with mistake/ complains/ failure* | Transactional leadership | Avolio, et al. (1999) |
| Success (B3) | Keeps track of all mistakes* | Transactional leadership | Avolio, et al. (1999) |
| Success (B3) | Directs my attention towards failures to meet standards* | Transactional leadership | Avolio, et al. (1999) |
| Success (B3) | Wait for things go to wrong before taking action* | Transactional leadership | Avolio, et al. (1999) |
| Success (B3) | Is excited about what needs to be accomplished | Transformational leadership | Avolio, et al. (1999) |
| Success (B3) | Expresses confidence on goal achievement | Transformational leadership | Avolio, et al. (1999) |
| Success (B3) | Entrepreneurial; seizes new opportunities in order to achieve goals. | Strategic Vision and Articulation | Conger, Kanungo, & Menon (2000) |
| Success (B3) | Readily recognizes new environmental opportunities that may facilitate achievement of organizational objectives. | Strategic Vision and Articulation | Conger, Kanungo, & Menon (2000) |
| Success (B3) | I stress being ahead of competing groups | Initiating Structure | Halpin & Stogdill (1962) |
| Success (B3) | I accept defeat in stride | Initiating Structure | Halpin & Stogdill (1962) |
| Success (B3) | I am working hard for a promotion | Initiating Structure | Halpin & Stogdill (1962) |
| Success (B3) | I enjoy the privileges of my position | Initiating Structure | Halpin & Stogdill (1962) |
| Success (B3) | I get what I ask for from my superiors | Initiating Structure | Halpin & Stogdill (1962) |
| Success (B3) | I urge the group to beat its previous record | Initiating Structure | Halpin & Stogdill (1962) |
| Success (B3) | I am working my way to the top | Initiating Structure | Halpin & Stogdill (1962) |
| Success (B3) | My boss reminds me of my past mistakes and failures* | Undermining | Tepper (2000) |
| Success (B3) | Getting ahead | Mediators | Judge, et al. (2004) |
| Inclusion (C1) | I give personal attention to others who seem rejected. | Individualized Consideration | Bass & Avolio (1992) |
| Inclusion (C1) | I get along well with the people above me | Consideration | Halpin & Stogdill (1962) |
| Inclusion (C1) | I keep the group working together as a team | Consideration | Halpin & Stogdill (1962) |
| Inclusion (C1) | I keep the group in good standing with higher authority | Consideration | Halpin & Stogdill (1962) |
| Inclusion (C1) | I settle conflicts when they occur in the group | Consideration | Halpin & Stogdill (1962) |
| Inclusion (C1) | I keep to myself* | Consideration | Halpin & Stogdill (1962) |
| Inclusion (C1) | I make sure that my part in the group is understood by the group members | Initiating Structure | Halpin & Stogdill (1962) |
| Inclusion (C1) | I help group members settle their differences | Consideration | Halpin & Stogdill (1962) |
| Inclusion (C1) | I maintain a closely knit group | Consideration | Halpin & Stogdill (1962) |
| Inclusion (C1) | My department manager spends the time to form quality relationships with departmental employees. | Emotional Healing | Ehrhart (2004) |
| Inclusion (C1) | My department manager creates a sense of community among departmental employees. | Building Community | Ehrhart (2004) |
| Inclusion (C1) | My department manager makes me feel like I work with him/her, not for him/her. | Altruistic Calling | Ehrhart (2004) |
| Inclusion (C1) | My boss does not allow me to interact with my coworkers* | Inequity | Tepper (2000) |
| Inclusion (C1) | As a leader I seek feedback to improve interactions with others | Self-awareness | Luthans & Avolio (2003) |
| Inclusion (C1) | This person believes that our organization needs to function as a community | Organizational stewardship | Barbuto & Wheeler (2006) |
| Inclusion (C1) | This person encourages me to have a community spirit in the workplace | Organizational stewardship | Barbuto & Wheeler (2006) |
| Inclusion (C1) | Getting along | Mediators | Judge, et al. (2004) |
| Caring (C2) | I make others feel good to be around me. | Idealized Influence | Bass & Avolio (1992) |
| Caring (C2) | I look out for the personal welfare of group members | Consideration | Halpin & Stogdill (1962) |
| Caring (C2) | Shows sensitivity for the needs and feelings of the other members in the organization. | Sensitivity to Member Needs | Conger, Kanungo, & Menon (2000) |
| Caring (C2) | Often expresses personal concern for the needs and feelings of other members in the organization. | Sensitivity to Member Needs | Conger, Kanungo, & Menon (2000) |
| Caring (C2) | Do you know where you stand with your leader (follower) . . .[and] do you usually know how satisfied your leader (follower) is with what you do? | Loyalty | Graen & Uhl-Bien (1995) |
| Caring (C2) | How would you characterize your working relationship with your leader (follower)? | Affect | Graen & Uhl-Bien (1995) |
| Caring (C2) | I do little things to make it pleasant to be a member of the group | Consideration | Halpin & Stogdill (1962) |
| Caring (C2) | I get my superiors to act for the welfare of the group members | Consideration | Halpin & Stogdill (1962) |
| Caring (C2) | I am friendly and approachable | Consideration | Halpin & Stogdill (1962) |
| Caring (C2) | Listens to what employees have to say. | Transparent and Open Communication | Brown, Treviño, & Harrison (2005) |
| Caring (C2) | Has the best interests of employees in mind. | Concern for Stakeholders | Brown, Treviño, & Harrison (2005) |
| Caring (C2) | My boss ridicules me* | Hostility | Tepper (2000) |
| Caring (C2) | My boss tells me my thoughts or feelings are stupid* | Hostility | Tepper (2000) |
| Caring (C2) | My boss gives me the silent treatment* | Hostility | Tepper (2000) |
| Caring (C2) | My boss expresses anger at me when he/she is mad for another reason* | Hostility | Tepper (2000) |
| Caring (C2) | My boss is rude to me* | Hostility | Tepper (2000) |
| Caring (C2) | As a leader I show I understand how specific actions impact others | Self-awareness | Luthans & Avolio (2003) |
| Caring (C2) | This person is one I would turn to if I had a personal trauma | Emotional healing | Barbuto & Wheeler (2006) |
| Caring (C2) | This person is good at helping me with my emotional issues | Emotional healing | Barbuto & Wheeler (2006) |
| Caring (C2) | This person is talented at helping me to heal emotionally | Emotional healing | Barbuto & Wheeler (2006) |
| Caring (C2) | This person is one that could help me mend my hard feelings | Emotional healing | Barbuto & Wheeler (2006) |
| Recognition (C3) | Others have complete faith in me. | Idealized Influence | Bass & Avolio (1992) |
| Recognition (C3) | I provide recognition/rewards when others reach their goals. | Contingent Reward | Bass & Avolio (1992) |
| Recognition (C3) | Others are proud to be associated with me. | Idealized Influence | Bass & Avolio (1992) |
| Recognition (C3) | Instills pride in me for being associated with her/him | Transformational leadership | Avolio, et al. (1999) |
| Recognition (C3) | Have my respect | Transformational leadership | Avolio, et al. (1999) |
| Recognition (C3) | I have enough confidence in my leader (follower) that I would defend and justify his or her decision if he or she were not present to do so. | Loyalty | Graen & Uhl-Bien (1995) |
| Recognition (C3) | I act as the spokesman of the group. | Initiating Structure | Halpin & Stogdill (1962) |
| Recognition (C3) | I maintain cordial relationship with superiors | Initiating Structure | Halpin & Stogdill (1962) |
| Recognition (C3) | I publicize the activities of the group | Consideration | Halpin & Stogdill (1962) |
| Recognition (C3) | I speak as a representative of the group | Initiating Structure | Halpin & Stogdill (1962) |
| Recognition (C3) | I speak for the group when visitors are present | Initiating Structure | Halpin & Stogdill (1962) |
| Recognition (C3) | My superiors act favorably on most of my suggestions | Initiating Structure | Halpin & Stogdill (1962) |
| Recognition (C3) | I represent the group at outside meetings | Initiating Structure | Halpin & Stogdill (1962) |
| Recognition (C3) | I am the leader of the group in name only* | Initiating Structure | Halpin & Stogdill (1962) |
| Recognition (C3) | I am easily recognized as the leader of the group | Initiating Structure | Halpin & Stogdill (1962) |
| Recognition (C3) | My boss puts me down in front of others* | Hostility | Tepper (2000) |
| Recognition (C3) | My boss doesn't give me credit for jobs requiring a lot of effort* | Inequity | Tepper (2000) |
| Recognition (C3) | My boss blames me to save himself/herself embarrassment* | Attribution of Blame | Tepper (2000) |
| Recognition (C3) | My boss makes negative comments about me to others* | Undermining | Tepper (2000) |
| Recognition (C3) | Influences others by developing mutual liking and respect. | Sensitivity to Member Needs | Conger, Kanungo, & Menon (2000) |
| Recognition (C3) | Perceived as leader-like | Emergence | Judge, et al. (2004) |
| Justice (D1) | I treat all group members as my equals | Consideration | Halpin & Stogdill (1962) |
| Justice (D1) | I refuse to explain my actions* | Consideration | Halpin & Stogdill (1962) |
| Justice (D1) | Makes fair and balanced decisions. | Fairness | Brown, Treviño, & Harrison (2005) |
| Ethics (D2) | Goes beyond self-interest for the good of staff | Transformational leadership | Avolio, et al. (1999) |
| Ethics (D2) | Talks only on most important values and beliefs | Transformational leadership | Avolio, et al. (1999) |
| Ethics (D2) | Considers moral & ethical consequences of decisions | Transformational leadership | Avolio, et al. (1999) |
| Ethics (D2) | Re-examines critical assumptions to question whether they are appropriate | Transformational leadership | Avolio, et al. (1999) |
| Ethics (D2) | Takes high personal risks for the sake of the organization. | Personal Risk | Conger, Kanungo, & Menon (2000) |
| Ethics (D2) | Often incurs high personal cost for the good of the organization. | Personal Risk | Conger, Kanungo, & Menon (2000) |
| Ethics (D2) | In pursuing organizational objectives, engages in activities involving considerable personal risk. | Personal Risk | Conger, Kanungo, & Menon (2000) |
| Ethics (D2) | Regardless of how much formal authority your leader (follower) has built into his or her position, what are the chances that your leader (follower) would use his or her power to help you solve problems in your work? | Contribution | Graen & Uhl-Bien (1995) |
| Ethics (D2) | Again, regardless of the amount of formal authority your leader (follower) has, what are the chances that he or she would “bail you out” at his or her expense? | Contribution | Graen & Uhl-Bien (1995) |
| Ethics (D2) | I ask that group members to follow standard rules and regulations | Initiating Structure | Halpin & Stogdill (1962) |
| Ethics (D2) | Disciplines employees who violate ethical standards. | Moral manager | Brown, Treviño, & Harrison (2005) |
| Ethics (D2) | Conducts his/her personal life in an ethical manner. | Moral person | Brown, Treviño, & Harrison (2005) |
| Ethics (D2) | Can be trusted. | Moral person | Brown, Treviño, & Harrison (2005) |
| Ethics (D2) | Discusses business ethics or values with employees. | Moral manager/Ethical guidance | Brown, Treviño, & Harrison (2005) |
| Ethics (D2) | Sets an example of how to do things the right way in terms of ethics. | Role Modeling through Visible Action/Ethical Guidance | Brown, Treviño, & Harrison (2005) |
| Ethics (D2) | Defines success not just by results but also the way that they are obtained. | Moral person | Brown, Treviño, & Harrison (2005) |
| Ethics (D2) | When making decisions, asks “what is the right thing to do?” | Moral Manager/Decision-making | Brown, Treviño, & Harrison (2005) |
| Ethics (D2) | My department manager holds employees to high ethical standards. | Organizational Stewardship | Ehrhart (2004) |
| Ethics (D2) | My department manager does what she or he promises to do. | Organizational Stewardship | Ehrhart (2004) |
| Ethics (D2) | My department manager encourages department employees to be involved in community service and volunteer activities outside of work. | Building Community | Ehrhart (2004) |
| Ethics (D2) | My department manager emphasizes the importance of giving back to the community. | Building Community | Ehrhart (2004) |
| Ethics (D2) | My boss breaks promises he/she makes* | Undermining | Tepper (2000) |
| Ethics (D2) | My boss lies to me* | Undermining | Tepper (2000) |
| Ethics (D2) | As a leader I know when it is time to reevaluate my position on important issues | Self-awareness | Luthans & Avolio (2003) |
| Ethics (D2) | As a leader I say exactly what I mean | Relational transparency | Luthans & Avolio (2003) |
| Ethics (D2) | As a leader I admit mistakes when they are made | Relational transparency | Luthans & Avolio (2003) |
| Ethics (D2) | As a leader I tell you the hard truth | Relational transparency | Luthans & Avolio (2003) |
| Ethics (D2) | As a leader I display emotions exactly in line with feelings | Relational transparency | Luthans & Avolio (2003) |
| Ethics (D2) | As a leader I solicit view that challenge my deeply held positions | Balanced processing | Luthans & Avolio (2003) |
| Ethics (D2) | As a leader I analyze all relevant data before coming to a decision | Balanced processing | Luthans & Avolio (2003) |
| Ethics (D2) | As a leader I listen carefully to different points of view before coming to conclusions | Balanced processing | Luthans & Avolio (2003) |
| Ethics (D2) | As a leader I demonstrate beliefs that are consistent with actions | Internalized moral perspective | Walumbwa et al. (2008) |
| Ethics (D2) | As a leader I make decisions based on my core values | Internalized moral perspective | Walumbwa et al. (2008) |
| Ethics (D2) | As a leader I ask you to take positions that support your core values | Internalized moral perspective | Walumbwa et al. (2008) |
| Ethics (D2) | As a leader I make difficult decisions based on high standards of ethical conduct | Internalized moral perspective | Walumbwa et al. (2008) |
| Ethics (D2) | This person puts my best interests ahead of his/her own | Altruistic calling | Barbuto & Wheeler (2006) |
| Ethics (D2) | This person does everything he/she can do to serve me | Altruistic calling | Barbuto & Wheeler (2006) |
| Ethics (D2) | This person sacrifices his/her own interests to meet my needs | Altruistic calling | Barbuto & Wheeler (2006) |
| Ethics (D2) | This person goes above and beyond the call of duty to meet my needs | Altruistic calling | Barbuto & Wheeler (2006) |
| Purpose (D3) | I provide appealing images about what we can do. | Inspirational Motivation | Bass & Avolio (1992) |
| Purpose (D3) | I help others find meaning in their work. | Inspirational Motivation | Bass & Avolio (1992) |
| Purpose (D3) | Specific importance of having a strong sense of purpose | Transformational leadership | Avolio, et al. (1999) |
| Purpose (D3) | Emphasizes important of group’s mission | Transformational leadership | Avolio, et al. (1999) |
| Purpose (D3) | Talks optimistically about future | Transformational leadership | Avolio, et al. (1999) |
| Purpose (D3) | Articulates a compelling vision | Transformational leadership | Avolio, et al. (1999) |
| Purpose (D3) | Provides inspiring strategic and organizational goals. | Strategic Vision and Articulation | Conger, Kanungo, & Menon (2000) |
| Purpose (D3) | Inspirational: able to motivate by articulating effectively the importance of what organizational members are doing. | Strategic Vision and Articulation | Conger, Kanungo, & Menon (2000) |
| Purpose (D3) | I speak from a strong inner conviction | Initiating Structure | Halpin & Stogdill (1962) |
| Purpose (D3) | I am an inspiring talker | Initiating Structure | Halpin & Stogdill (1962) |
| Purpose (D3) | This person believes that the organization needs to play a moral role in society | Organizational stewardship | Barbuto & Wheeler (2006) |
| Purpose (D3) | This person sees the organization for its potential to contribute to society | Organizational stewardship | Barbuto & Wheeler (2006) |
| Purpose (D3) | This person is preparing the organization to make a positive difference in the future | Organizational stewardship | Barbuto & Wheeler (2006) |
| Purpose (D3) | Providing meaning | Mediators | Judge, et al. (2004) |

Sources: Avolio et al., 1999; Barbuto and Wheeler, 2006; Brown et al., 2005; Conger et al., 2000; Ehrhart, 2004; Graen and Uhl-Bien, 1995; Halpin and Stogdill, 1962; Judge et al., 2004; Luthans and Avolio, 2003; Tepper, 2000; and Walumbwa et al., 2008.
